# E-Selectin/AAV Gene Therapy Promotes Myogenesis and Skeletal Muscle Recovery in a Mouse Hindlimb Ischemia Model

**DOI:** 10.1155/2023/6679390

**Published:** 2023-05-19

**Authors:** Antoine J. Ribieras, Yulexi Y. Ortiz, Yan Li, Nga T. Le, Carlos T. Huerta, Francesca A. Voza, Hongwei Shao, Roberto I. Vazquez-Padron, Zhao-Jun Liu, Omaida C. Velazquez

**Affiliations:** ^1^Division of Vascular Surgery, DeWitt Daughtry Family Department of Surgery, University of Miami Miller School of Medicine, Miami, FL 33136, USA; ^2^Vascular Biology Institute, University of Miami Miller School of Medicine, Miami, FL 33136, USA

## Abstract

The response to ischemia in peripheral artery disease (PAD) depends on compensatory neovascularization and coordination of tissue regeneration. Identifying novel mechanisms regulating these processes is critical to the development of nonsurgical treatments for PAD. E-selectin is an adhesion molecule that mediates cell recruitment during neovascularization. Therapeutic priming of ischemic limb tissues with intramuscular E-selectin gene therapy promotes angiogenesis and reduces tissue loss in a murine hindlimb gangrene model. In this study, we evaluated the effects of E-selectin gene therapy on skeletal muscle recovery, specifically focusing on exercise performance and myofiber regeneration. C57BL/6J mice were treated with intramuscular E-selectin/adeno-associated virus serotype 2/2 gene therapy (E-sel/AAV) or LacZ/AAV2/2 (LacZ/AAV) as control and then subjected to femoral artery coagulation. Recovery of hindlimb perfusion was assessed by laser Doppler perfusion imaging and muscle function by treadmill exhaustion and grip strength testing. After three postoperative weeks, hindlimb muscle was harvested for immunofluorescence analysis. At all postoperative time points, mice treated with E-sel/AAV had improved hindlimb perfusion and exercise capacity. E-sel/AAV gene therapy also increased the coexpression of MyoD and Ki-67 in skeletal muscle progenitors and the proportion of Myh7^+^ myofibers. Altogether, our findings demonstrate that in addition to improving reperfusion, intramuscular E-sel/AAV gene therapy enhances the regeneration of ischemic skeletal muscle with a corresponding benefit on exercise performance. These results suggest a potential role for E-sel/AAV gene therapy as a nonsurgical adjunct in patients with life-limiting PAD.

## 1. Introduction

Peripheral artery disease (PAD) is the manifestation of systemic atherosclerosis in the extremities. PAD affects 8.5 million people in the United States and more than 200 million worldwide [1]. Symptomatic patients with PAD typically present with exertional calf pain known as intermittent claudication [1–3]. The pathophysiology of intermittent claudication in PAD is related to both impaired perfusion and skeletal muscle dysfunction [4, 5]. Histologically, decreased size and abnormal morphology of myofibers correlate with functional impairments such as calf muscle strength and walking distance [6]. Neovascularization depends on the recruitment of endothelial progenitor cells (EPCs), remodeling of the tissue microenvironment, and coordination of endothelial sprouting [7]. On the other hand, skeletal muscle regeneration requires the activation, proliferation, and differentiation of muscle stem cells known as satellite cells. Nevertheless, there is considerable overlap between these two processes during the regeneration of ischemic muscle.

Several growth factors have been shown to play a role in the regulation of both neovascularization and skeletal muscle regeneration. Vascular endothelial growth factor (VEGF) is secreted in response to tissue hypoxia and primarily drives endothelial tip cell migration via Notch signaling and increased expression of Notch ligands such as delta-like 4 (DLL4) [8, 9]. However, there is also VEGF-mediated crosstalk between the vascular and muscle stem cell niche. Satellite cell-derived VEGF regulates the proximity of blood vessels to satellite cells while endothelial cells maintain satellite cell self-renewal [10]. VEGF also promotes the fusion of myogenic cells into myotubes and protects against apoptosis [11]. Similarly, fibroblast growth factor (FGF) and hepatocyte growth factor (HGF) act synergistically as mitogens for activated satellite cells, and HGF specifically stimulates the early entry of satellite cells into the cell cycle [12]. The angiopoietin family of growth factors, specifically Ang-1, has also been implicated in both remodeling of blood vessels and myoblast differentiation [13, 14]. In practice, however, clinical trials of FGF, HGF, and VEGF have shown modest benefit for improving limb perfusion and wound healing in patients with PAD and CLTI [15–20].

E-selectin (CD62E) is a cell adhesion molecule that is expressed by activated endothelium in response to tissue hypoxia and injury. E-selectin mediates the recruitment of EPCs to areas of ischemia and wound healing where these cells contribute to neovascularization [21]. Thus, augmentation of E-selectin levels in ischemic tissue may be an alternative strategy for therapeutic angiogenesis. To this end, we have previously shown in preclinical models that E-selectin/adeno-associated virus gene therapy (E-sel/AAV) enhances the recruitment of EPCs and modulates the angiogenic and inflammatory gene expression profiles of ischemic muscle with a corresponding improvement in perfusion, wound healing, and tissue loss [22–24]. To better characterize this gene therapy, this study focuses on the effects of E-sel/AAV on the regeneration and functional recovery of ischemic skeletal muscle.

## 2. Materials and Methods

### 2.1. Production of Adeno-Associated Virus Vectors

Murine E-selectin and LacZ genes were inserted into multiple cloning sites in the pZac vector. After confirmation by Sanger sequencing, E-selectin/pZac and LacZ/pZac plasmids were sent to the University of North Carolina Gene Therapy Vector Core where AAV serotype 2/2 was prepared by three-plasmid transfection into HEK293 cells [25]. Quality assurance and control testing were performed by polymerase chain reaction (PCR) quantification of genomes and infectivity titer.

### 2.2. Gene Therapy Administration

Animal experiments were performed in C57BL/6J male and female mice (000664, Jackson Laboratory, Bar Harbor, ME) aged 10-12 weeks old. All protocols were approved by the University of Miami Institutional Animal Care and Use Committee (22-096). To account for the lag time between AAV injections and tissue transgene expression, gene therapy was administered 4 and 2 days prior to and on the day of surgery. Total dose per mouse was 1 × 10^11^ viral genome divided across the 3 days and diluted in 100 *μ*L phosphate-buffered saline (PBS) each day. Mice were anesthetized with inhaled isoflurane 1.5-2% and oxygen at 2 L/min and administered intramuscular (IM) injection of either E-sel/AAV or LacZ/AAV (*N* = 20 per group, 10 females per group) into five sites in the left adductor group (2), lateral thigh (1), and medial (1) and lateral (1) gastrocnemius.

### 2.3. Induction of Hindlimb Ischemia

**Figure 1:**
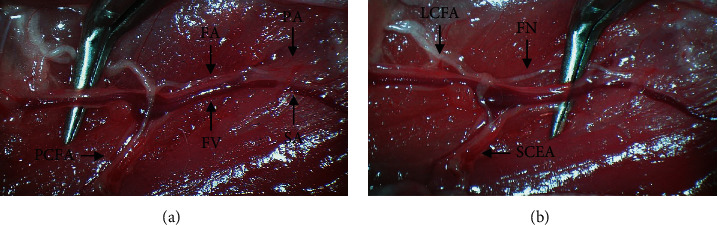
Intraoperative images of the murine hindlimb vasculature. Coagulation of the femoral artery (FA) and vein (FV) is performed (a) proximally and (b) distally with preservation of the femoral nerve (FN) as indicated by the forceps. PCFA: proximal caudal femoral artery; LCFA: lateral circumflex femoral artery; PA: popliteal artery; SA: saphenous artery; SCEA: superficial caudal epigastric artery.

Hindlimb ischemia was induced according to a previously described protocol [26]. Mice were anesthetized by intraperitoneal (IP) injection of ketamine (80 mg/kg) and xylazine (5 mg/kg). After hair removal, the left groin was prepped with chlorohexidine. A 1 cm incision was made in the left groin, and the inguinal fat was dissected from the inguinal ligament. The femoral sheath was entered, and the femoral nerve was isolated from the femoral vessels. The femoral artery and vein were coagulated with an electrocautery device just proximal to the lateral circumflex femoral artery (Figure 1(a)) and just proximal to the saphenopopliteal bifurcation (Figure 1(b)). Hemostasis was obtained, and the wound was closed with a 5-0 absorbable suture.

### 2.4. Laser Doppler Perfusion Imaging

Hindlimb perfusion was measured using a moorLDI laser Doppler perfusion imaging (LDPI) device and quantified in version 5 software (Moor Instruments, Wilmington, DE). To acquire images, mice (*N* = 20 per group) were anesthetized with inhaled isoflurane 1.5-2% and oxygen at 2 L/min and placed in a prone position on a black foam mat. Body temperature was maintained with a heating pad. After scanning, the perfusion index was calculated as the ratio of mean flux values from the left/ischemic relative to the right/non-ischemic hindlimb.

### 2.5. Grip Strength Testing

**Figure 2:**
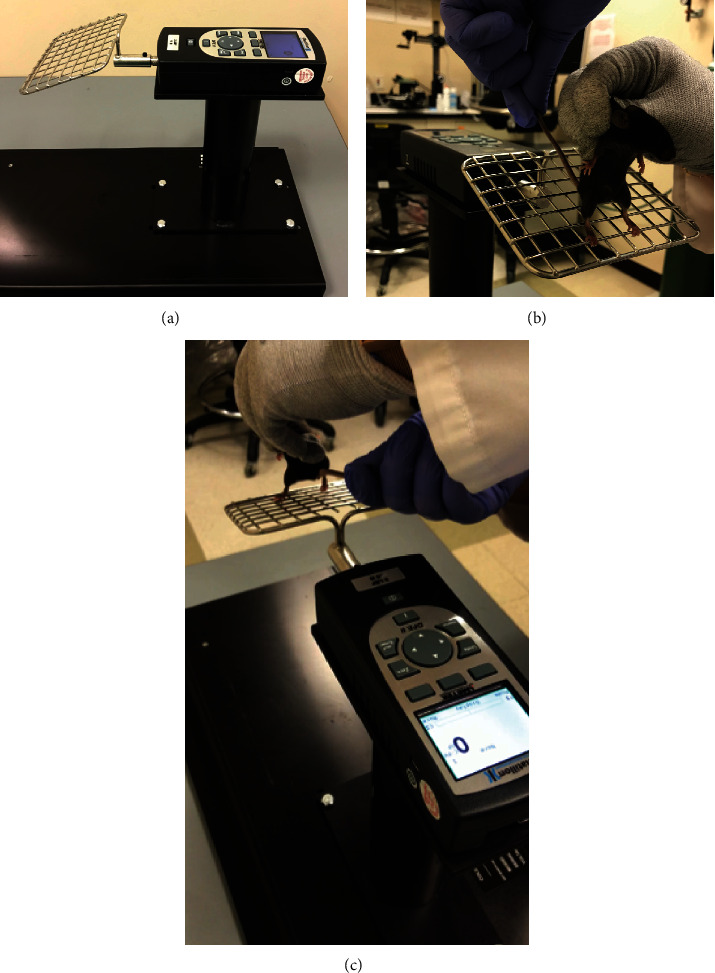
(a) Grip strength meter with (b, c) positioning of the mouse on grid assembly.

Grip strength was measured using a grip strength meter (Columbus Instruments, Columbus, OH) fitted with a mesh grid assembly and set to peak compression mode (Figure 2(a)). Mice (*N* = 20 per group) were held by the dorsal skin and placed on the grid assembly such that only the hindlimbs grasped the grid (Figure 2(b)). The animal was then gently pulled backwards towards the grip strength meter, and the maximal compression force was recorded in gram-force (gf) units (Figure 2(c)). Three separate readings were obtained during each session. Results are reported as the mean of three readings and the best reading normalized to body weight at the time of testing.

### 2.6. Treadmill Exhaustion Testing

Mice (*N* = 8 per group) were trained to run on an Exer 3/6 treadmill (Columbus Instruments) during 4 sessions across the 2 weeks prior to surgery. The treadmill was set at a 10° incline with shocks at 1 Hz. For training sessions, mice walked on the treadmill with at a speed of 10 m/min for 10 minutes and then 15 m/min for 5 minutes. For exhaustion testing, mice were allowed to warm up with the treadmill speed set at 5 m/min and then ramped up by 1 m/min^2^. Distance recording was started when the speed reached 10 m/min. After 5 minutes, treadmill speed was increased to 15 m/min, and then by 3 m/min every 5 minutes until a maximum speed of 30 m/min. Exhaustion was defined as 40 shocks, after which they were disabled, and total walking distance was recorded.

### 2.7. Immunofluorescence Assays

Mice were euthanized on POD 21 for harvesting of the left and right adductor and gastrocnemius muscles. Tissue samples were fixed in 10% formalin, embedded in paraffin, and sectioned. Slides were deparaffinized per standard protocol, and antigen retrieval was performed in EDTA buffer (pH 9.0) at 120°C for 10 minutes. Slides were washed in distilled water and permeabilized with 0.25% Triton-X100 TBS for 15 minutes. Tissue was incubated with Protein Block (ab64226, Abcam, Cambridge, United Kingdom) for 1 hour. Slides were then incubated overnight at 4°C with primary antibodies (5 *μ*g/mL) for E-selectin (148802, BioLegend, San Diego, CA), MyoD (NBP1-54153, Novus Biologicals, Littleton, CO), Ki-67 (SC-7846, Santa Cruz Biotechnology, Dallas, TX), laminin (NBP2-44751, Novus), and Myh7 (NBP2-94079, Novus) followed by Alexa Fluor 488 donkey anti-rabbit IgG (A21206, Invitrogen, Waltham, MA), Alexa Fluor 488 goat anti-mouse (A11029), Alexa Fluor 594 chicken anti-goat IgG (A21468, Invitrogen), or Alexa Fluor 594 goat anti-rat IgG (A11007, Invitrogen) as appropriate (2 *μ*g/mL). Slides were imaged at 20x magnification with a Zeiss Axio Observer inverted microscope (ZEISS, Oberkochen, Germany). For each stain, a blinded observer acquired at least 4 images from 4 sections per mouse (*N* = 5 per group) and performed cell counting in Fiji.

### 2.8. PCR Quantification of E-Sel Transgene Expression

Muscle harvested on POD 21 was homogenized in TRIzol reagent (15596018, Invitrogen/Thermo Fisher Scientific). Total RNA was extracted and reverse transcribed using RT^2^ First Strand Kit (Qiagen, Venlo, Netherlands). Real-time reverse transcription quantitative PCR (RT-qPCR) was performed using RT^2^ SYBR Green qPCR Mastermix (330500, Qiagen) and primers for E-sel (Sele, NM_011345, assay ID Mm.PT.58.11296882, primers 5′-GTCATCCTGTAACTTCACCTGT-3′ and 5′-CGCAGATAAGGCTTCACAC-3′) and Rplp0 (NM_007475, assay ID Mm.PT.58.43894205, primers 5′-TTATAACCCTGAAGTGCTCGAC-3′ and 5′-CGCTTGTACCCATTGATGATG-3′) as housekeeping gene (Integrated DNA Technologies, Coralville, IA). Assays were performed in duplicate (*N* = 4 per group) and analyzed using the ΔCt method (2^−ΔΔCt^) method.

### 2.9. Statistics

Statistical analyses were performed using GraphPad Prism (version 9.0.1, GraphPad Software, San Diego, CA). All continuous data were normally distributed by the Shapiro–Wilk test and compared using Student's *t*-test. Data are presented as mean ± standard error (SEM) with statistical significance set as *P* < .05.

## 3. Results

### 3.1. E-Sel/AAV Induces High-Level Transgene Expression in Skeletal Muscle

**Figure 3:**
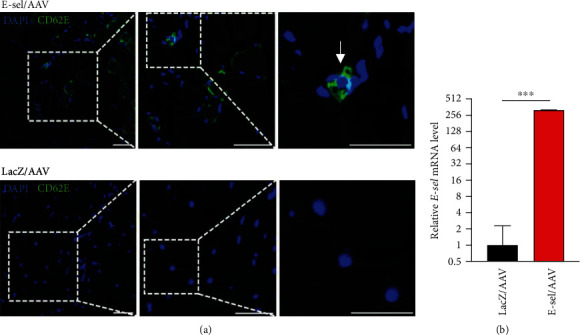
E-sel/AAV induces high-level transgene expression. (a) Immunofluorescence pattern of E-selectin (CD62E) expression in ischemic muscle with some CD62E^+^ cells forming capillaries (arrows). (b) E-sel mRNA levels in ischemic muscle are 322-fold higher three weeks after treatment with E-sel/AAV compared to LacZ/AAV (*N* = 4 per group). Scale bars represent 50 *μ*m. Data are presented as mean ± SEM where ^∗∗∗^*P* < .001.

To account for the lag time between AAV injection and tissue transgene expression, which normally takes 2-4 days, gene therapy was administered 4 and 2 days preoperatively and immediately prior to surgery. A total of 1 × 10^11^ viral genomes divided across 3 doses of either E-sel/AAV or LacZ/AAV control vector were administered to 5 sites in the left thigh adductor and calf muscles. Hindlimb ischemia was then induced by left femoral artery and vein coagulation. Expression of E-selectin after treatment with E-sel/AAV was assessed by immunofluorescence (Figure 3(a)) and qRT-PCR (Figure 3(b)). In muscle treated with E-sel/AAV, E-selectin was primarily concentrated at the plasma membrane of some muscle fibers and in cells located between muscle fibers organizing into capillaries. Quantitatively, E-sel mRNA levels were 322-fold higher in ischemic muscle three weeks after treatment with E-sel/AAV compared to LacZ/AAV, indicating high-level and durable transgene expression with this vector.

### 3.2. E-Sel/AAV Improves Reperfusion of Ischemic Hindlimb

**Figure 4:**
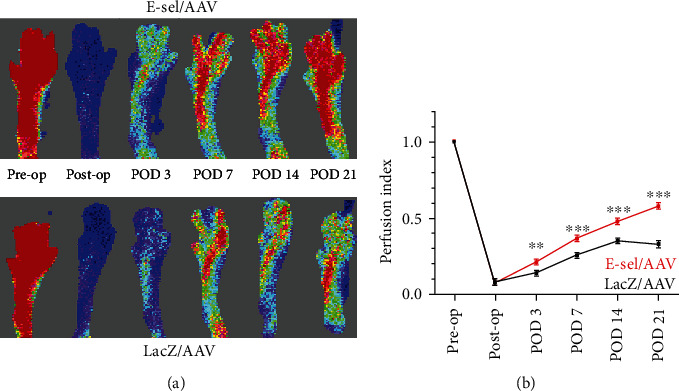
E-sel/AAV enhances reperfusion of ischemic muscle. (a) The representative laser Doppler perfusion images with (b) quantification of perfusion indices demonstrating improved recovery of footpad perfusion in mice treated with E-sel/AAV compared to LacZ/AAV (*N* = 20 per group). Data are presented as mean ± SEM, where ^∗∗^*P* < .01 and ^∗∗∗^*P* < .001.

After femoral artery coagulation, both E-sel/AAV- and LacZ/AAV-treated mice experienced a similar reduction in hindlimb perfusion (0.08 ± 0.01 vs. 0.08 ± 0.01, *P* = .64) (Figures 4(a) and 4(b)). Perfusion then progressively improved in both groups but was significantly enhanced by E-sel/AAV starting on POD 3 (0.21 ± 0.02 vs. 0.14 ± 0.01, *P* = .002) and at all time points through POD 21 (0.58 ± 0.02 vs. 0.33 ± 0.02, *P* < .001).

### 3.3. E-Sel/AAV Enhances Recovery of Ischemic Hindlimb Grip Strength and Exercise Capacity

**Figure 5:**
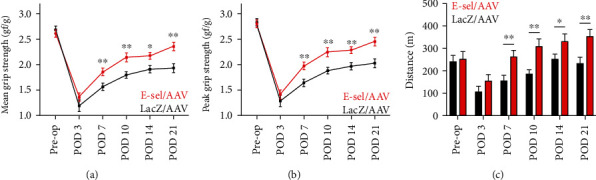
E-sel/AAV enhances functional recovery of ischemic muscle. (a) Mean and (b) peak postoperative hindlimb grip strengths are increased in mice treated with E-sel/AAV compared to LacZ/AAV (*N* = 20 per group). (c) Maximal distance traveled on treadmill exhaustion testing is increased in mice treated with E-sel/AAV compared to LacZ/AAV (*N* = 8 per group). Data are presented as mean ± SEM, where ^∗^*P* < .05 and ^∗∗^*P* < .01.

To assess functional recovery, we tested both hindlimb grip strength and aerobic exercise capacity by treadmill exhaustion. In both groups, hindlimb grip strength was acutely impaired after femoral artery coagulation and then gradually recovered in parallel with reperfusion. However, starting on POD 7 (mean grip strength 1.89 ± 0.08 vs. 1.57 ± 0.07 gf/g, *P* = .009; peak grip strength 1.97 ± 0.08 vs. 1.64 ± 0.08 gf/g, *P* = .006) and through POD 21 (mean grip strength 2.36 ± 0.08 vs. 1.93 ± 0.09 gf/g, *P* = .001; peak grip strength 2.45 ± 0.08 vs. 2.03 ± 0.09, *P* = .001), both mean and peak grip strengths were significantly greater in mice treated with E-sel/AAV compared to LacZ/AAV control vector (Figures 5(a), 5(b)). Similarly, recovery of exercise capacity on treadmill exhaustion testing was improved in E-sel/AAV-treated mice compared to controls starting on POD 7 (264 ± 26 vs. 157 ± 23 m, *P* = .009) and through POD 21 (354 ± 27 vs. 232 + 30 m, *P* = .009) (Figure 5(c)).

### 3.4. E-Sel/AAV Increases Activation of Myogenic Precursors

**Figure 6:**
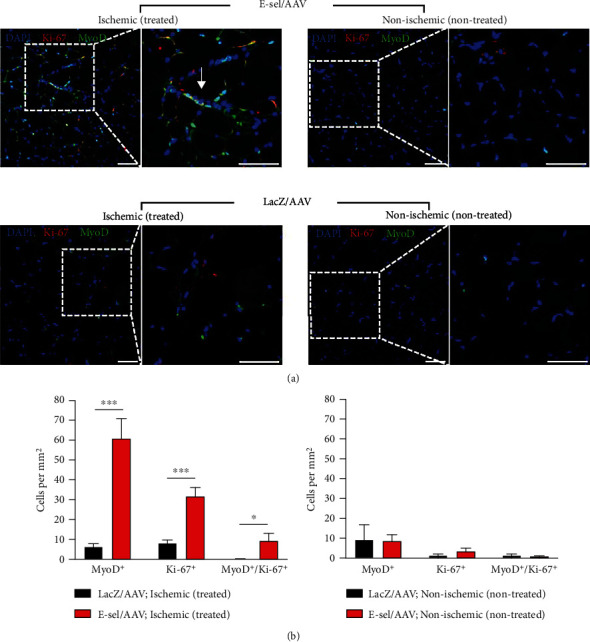
E-sel/AAV enhances proliferation of myogenic precursors in ischemic muscle. (a) Representative immunofluorescence images and (b) quantification of MyoD and Ki-67 expression demonstrating increased number of MyoD^+^/Ki-67+ myogenic precursors (white arrow) in ischemic gastrocnemius muscle treated with E-sel/AAV compared to LacZ/AAV (*N* = 5 per group). In comparison, few MyoD^+^ or Ki-67^+^ cells are identified in non-treated, non-ischemic muscle from either group. Scale bars represent 50 *μ*m. Data are presented as mean ± SEM, where ^∗^*P* < .05 and ^∗∗∗^*P* < .001.

To determine whether treatment with E-sel/AAV affected the activation of skeletal muscle precursors, we performed immunofluorescence staining for the myogenic differentiation marker MyoD and proliferation marker Ki-67 (Figure 6(a)). On POD 21, ischemic calf muscle treated with E-sel/AAV demonstrated an increased number of MyoD^+^ cells compared to that treated with LacZ/AAV control vector (61.0 ± 9.9 vs. 6.2 ± 1.6 cells/mm^2^, *P* < .001) (Figure 6(b)). Similarly, there was an increased number of Ki-67^+^ cells in the E-sel/AAV-treated muscle compared to control (31.8 ± 4.3 vs. 8.0 ± 1.4 cells/mm^2^, *P* < .001). To compare the number of proliferating myogenic precursors, we then counted the number of cells costaining for both MyoD and Ki-67. As with the individual stains, there was also a greater number of MyoD^+^/Ki-67^+^ cells in ischemic muscle treated with E-sel/AAV compared to LacZ/AAV control vector (9.4 ± 3.6 vs. 0.1 ± 0.1 cells/mm^2^, *P* = .027). In contrast, there were few cells expressing MyoD (8.9 ± 2.9 vs. 9.0 ± 8.0 cells/mm^2^, *P* = .99), Ki-67 (3.5 ± 1.6 vs. 1.3 ± 0.7 cells/mm^2^, *P* = .29), or both MyoD and Ki-67 (0.4 ± 0.4 vs. 1.3 ± 0.7 cells/mm^2^, *P* = .29) in non-ischemic non-treated gastrocnemius muscle from both E-sel/AAV and LacZ/AAV groups (0.8 ± 0.5 vs. 1.3 ± 0.7 cells/mm^2^, *P* = .78). These findings indicate that at baseline, normal skeletal muscle has minimal regenerative activity. With tissue ischemia, however, skeletal muscle progenitor cells are activated and proliferate. This response can be significantly potentiated by E-sel/AAV gene therapy.

### 3.5. E-Sel/AAV Is Associated with Increased Myh7^+^ Myofiber Differentiation

**Figure 7:**
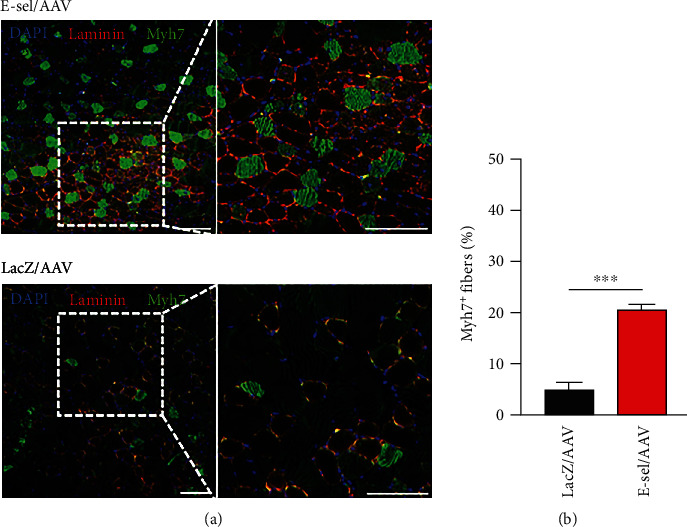
E-sel/AAV is associated with increased Myh7^+^ myofibers in regenerated skeletal muscle. (a) Representative immunofluorescence images and (b) quantification demonstrating increased proportion of Myh7^+^ myofibers in ischemic gastrocnemius muscle treated with E-sel/AAV compared to LacZ/AAV (*N* = 5 per group). Scale bars represent 50 *μ*m. Data are presented as mean ± SEM, where ^∗∗∗^*P* < .001.

We then assessed whether treatment with E-sel/AAV influenced the relative distribution of myofiber type in regenerating muscle. Specifically, we performed immunofluorescence staining for myosin heavy peptide 7 (Myh7), the myosin heavy chain isomer expressed in type I or slow-twitch myofibers (Figure 7(a)). Under normal conditions, Myh7 is not expressed in mouse gastrocnemius. In ischemic gastrocnemius, however, we found that treatment with E-sel/AAV gene therapy increased the proportion of Myh7^+^ fibers compared to LacZ/AAV control vector (21.0 ± 0.7% vs. 4.9 ± 1.5%, *P* < .001) (Figure 7(b)).

## 4. Discussion

In this study, we use an AAV vector to therapeutically increase E-selectin expression in ischemic mouse hindlimb muscle. First, we confirm the efficacy of AAV vector for high-level *in vivo* transgene expression. We then demonstrate the benefit of E-sel/AAV gene therapy for improving ischemic hindlimb reperfusion and functional recovery. In prior work, we used a FVB mice to create a hindlimb gangrene model and showed that E-sel/AAV can help restore blood flow and reduce severity of tissue loss [24]. In contrast, the C57BL/6 mice used in this study are more resistant to ischemia than FVB and even more so than BALB/c strains [27, 28]. As such, C57BL/6 mice do not develop toe or foot necrosis after femoral artery coagulation, which allowed us to test hindlimb grip strength as a novel endpoint in addition to treadmill exercise capacity.

Having observed a benefit of E-sel/AAV on functional recovery of skeletal muscle, we then sought to determine how E-selectin overexpression might affect myofiber regeneration. Adult skeletal muscle regeneration is driven by activation of muscle stem cells known as satellite cells. Satellite cells reside between the muscle sarcolemma and basal lamina and characteristically express the transcription factor Pax7 [29, 30]. Muscle injury activates the normally quiescent satellite cells to enter the cell cycle and then divide asymmetrically generating a progeny of committed precursor myoblasts while maintaining a self-renewing pool of satellite cells [31–33]. The differentiation of satellite cells into myoblasts and myofibers is coordinated by myogenic regulatory factors (MRFs) which comprise a family of basic helix-loop-helix transcription factors including MyoD, Myf5, myogenin, and MRF4 [34]. Sequential expression of MyoD and Myf5 coincides with satellite cell activation and proliferation and is required for myotube fusion and expression of myosin heavy chain (MyHC) [35, 36]. Thus, our finding that E-sel/AAV increased the number of cells expressing both MyoD and the proliferation marker Ki-67 suggests that this gene therapy may enhance myogenesis by increasing proliferation of muscle progenitor cells.

In addition to enhancing proliferation of skeletal muscle cell precursors, we found that E-selectin overexpression was associated with an increased proportion of Myh7^+^ myofibers. The Myh7 gene codes for MyHC-*β*/slow and is preferentially expressed in type I or slow-twitch oxidative fibers in the heart and skeletal muscle [37]. Type I fibers are adapted for endurance and aerobic metabolism and have greater mitochondrial and myoglobin content than fast-twitch glycolytic-oxidative (IIA, Myh2) and glycolytic (IIB/IIX, Myh4/Myh1) fibers. Whereas the distribution of fiber type varies across species and muscle group, fiber-type switching can be induced to a varying extent by activity and metabolic changes. In mice, the calcineurin-nuclear factor of activated T cells (NFAT) signaling cascade has been implicated in activity-dependent fast-to-slow fiber-type switching via increased expression of myoglobin [38] and enzymes responsible for mitochondrial oxidative phosphorylation and lipid metabolism [39, 40]. In human patients, chronic ischemia leads to preferential denervation and oxidative damage to type II fibers which leads to muscle weakness and exercise impairment [41, 42]. In contrast, we observed that treatment with E-sel/AAV in a mouse hindlimb ischemia model increased the proportion of type I fibers but also improved limb perfusion and function. This discrepancy may be explained by differences in muscle fiber distribution in human gastrocnemius which contains a mix of type I and type II fibers compared to mouse gastrocnemius which predominantly consists of type II fibers.

The mechanisms by which E-selectin overexpression affects skeletal muscle regeneration remain to be elucidated. E-selectin primarily mediates rolling and extravasation of circulating neutrophils and monocytes during inflammatory and thrombotic processes, as well as trafficking of bone marrow-derived EPCs to areas of ischemia for angiogenesis. Thus, E-sel/AAV may first enhance skeletal muscle regeneration indirectly by improving blood flow and nutrient delivery to ischemic tissue. However, E-selectin signaling has also been directly implicated in skeletal muscle homeostasis. For example, exercise can induce expression of endothelial cell adhesion molecules (CAMs) such as intercellular CAM 1 (ICAM-1), vascular CAM 1 (VCAM-1), and E-selectin in human skeletal muscle [43]. E-selectin can then induce mitogen-activated protein kinase (MAPK) signaling in cultured endothelial cells [44]. Downstream, activation of extracellular signal-related kinase 1/2 (ERK1/2) has been shown to not only stimulate arteriogenesis in synectin-deficient and atherosclerotic mice [45] but also induce type I/slow-twitch fiber-type switching which protects against muscle damage in a mouse dystrophy model [46].

Importantly, AAV does not readily transduce quiescent satellite cells [47]. Whereas ischemia may render satellite cells more receptive to transduction with AAV, this has not been demonstrated previously. More likely, the observed effects of E-sel/AAV on activation of myogenic precursors are due to paracrine signaling from other resident or recruited cells. Recently, we showed that E-sel/AAV gene therapy can modulate the angiogenic and inflammatory gene expression profile of ischemic muscle. Most notably, E-sel/AAV upregulated a number of angiogenic factors including interleukin 6 (IL-6), tumor necrosis factor *α* (TNF-*α*), and monocyte chemoattractant protein 1 (MCP-1) [24]. These same factors are also expressed by satellite cells in response to muscle injury [48]. IL-6 is an essential regulator of skeletal muscle hypertrophy in response to muscle lengthening [49, 50] and promotes satellite cell proliferation via autocrine and paracrine signaling via janus kinase (JAK)/signal transducer and activator of transcription (STAT). Macrophage chemoattractant protein 1 (MCP-1, Ccl2), on the other hand, mediates the recruitment of monocytes to ischemic tissue [51]. Inflammatory (M1) macrophages are the dominant cell population up to 21 days after ischemic insult and play a key role in both collateral vessel formation and skeletal muscle regeneration. Therapeutic administration of M1 macrophages can increase myofiber size, decrease fibrosis, and increase contractile force in ischemic muscle [52]. While this study did not assess inflammation, we previously found no difference in infiltration of Mac-2^+^ macrophages [23] or CD3^+^ T cells [24] in muscle treated with E-sel/AAV compared to LacZ/AAV. Other cell types such as mesenchymal stem cells (MSCs) have been shown to interact with satellite cells which can then induce MSC myogenic commitment [53].

There are limitations to the present study. Despite the widespread use of mouse hindlimb ischemia models in preclinical research, surgical disruption of the femoral artery is an acute ischemic insult rather than the chronic occlusive process of atherosclerosis in PAD. Moreover, experiments in young, healthy animals fail to account for various comorbidities such as hypertension, diabetes, and hyperlipidemia that patients with PAD often present with. Nevertheless, this study demonstrates that E-sel/AAV gene therapy can modulate the physiological response to ischemia. Future studies will be aimed at identifying the precise mechanisms of E-selectin-mediated activation of muscle progenitor cells and fiber-type switching, as well as assessing the *in vitro* effects of E-selectin overexpression on skeletal muscle cell proliferation, gene expression, and metabolism.

## 5. Conclusions

This study confirms the efficacy of intramuscular E-sel/AAV gene therapy for therapeutic angiogenesis in a mouse hindlimb ischemia model. In addition to improving muscle perfusion, E-sel/AAV enhances proliferation of myogenic precursors and is associated with increased proportion of type I/slow-twitch myofibers in regenerating ischemic muscle. Altogether, these effects correlate with improved exercise capacity and suggest a potential role for E-sel/AAV gene therapy as a nonsurgical adjunct for patients with life-limiting PAD.

## Data Availability

The data used to support the findings of this study are available from the corresponding author upon request.
